# Barriers to Optimal Acute Management of Stroke: Perspective of a Stroke Center in Mexico City

**DOI:** 10.3389/fneur.2021.690946

**Published:** 2021-12-07

**Authors:** Vanessa Cano-Nigenda, Enrique Castellanos-Pedroza, Diana Manrique-Otero, Beatriz Méndez, María Fernanda Menéndez-Manjarrez, Roberto Toledo-Treviño, Miguel Calderón, Antonio Arauz

**Affiliations:** ^1^Stroke Clinic, Instituto Nacional de Neurología y Neurocirugía Manuel Velasco Suárez, Mexico City, Mexico; ^2^Department of Endovascular Therapy, Instituto Nacional de Neurología y Neurocirugía Manuel Velasco Suárez, Mexico City, Mexico

**Keywords:** stroke, barriers, developing country, low-and middle-income countries, public health policies

## Abstract

**Background:** Stroke is a leading cause of death and disability worldwide, particularly in low- and middle-income countries. We aimed to identify the main barriers to optimal acute management of stroke in a referral center.

**Methods:** Demographic data was collected from patients assessed with acute stroke in the emergency department of the Instituto Nacional de Neurología y Neurocirugía (INNN) from January to June 2019. Additionally, a telephone interview was conducted with patients/primary caregiver to know which they considered the main reason for the delay in arrival at INNN since the onset of stroke.

**Results:** 116 patients were assessed [age 65 ± 15 years, 67 (57.8%) men]. Patients consulted other facilities prior to arrival at INNN in 59 (50.9%) cases (range of hospitals visited 1–4), 83 (71.6%) arrived in a private car, with prenotification in only 4 (3.4%) of the total sample. The mean onset-to-door time was 17 h (45 min−10 days). Telephone interviews were done in 61 patients/primary caregivers, stating that they consider the multiple evaluations in other facilities [*n* = 26/61 (42.6%)] as the main reason for delay in arrival at the ED, followed by ignorance of stroke symptoms and treatment urgency [*n* = 21/61 (34.4%)].

**Conclusion:** In this small, retrospective, single center study, the main prehospital barrier to optimal acute management of stroke in a developing country is multiple medical evaluations prior to the patient's transport to a specialized stroke hospital, who mostly arrived in a private car and without prenotification. These barriers can be overcome by strengthening public education and improving patient transfer networks and telemedicine.

## Introduction

Stroke remains the second leading cause of disability and death worldwide (1), particularly in low- and middle-income countries (LMICs), where most of the stroke burden occurs (2). The prospective data base from the National Institute of Neurology and Neurosurgery-Stroke Registry (NINN-SR), the largest hospital-based registry in Latin America, which included information on 4,481 strokes, showed a mortality rate of 24.5% and poor outcomes [modified Rankin scale (mRs) ≥ 3] in 56.2% of patients, mainly due to cerebral hemorrhage (3). In general, the mortality rate of stroke has been cut in half in high-income countries but reduced by only 15% in LMICs (4). In ischemic stroke (IS), the frequency of intravenous thrombolysis (IVT) use in Mexican hospitals is <10%, mainly because patients continue to arrive outside the therapeutic window (5).

The quality and quantity of stroke care is not homogeneous in developing countries. As observed in previous studies, there are multiple barriers at different levels of care, including at the patient level (sociocultural, stroke education, and financial considerations), in the healthcare system (inadequate stroke care protocol and a limited number of stroke team members), at the healthcare professional level (low collaboration, limited and outdated knowledge of stroke) and regarding national health policies (6–8).

The objective of this study is to evaluate the barriers and limitations to optimal acute management of stroke in a developing country.

## Materials and Methods

This is a retrospective analysis of the prospective cohort of the Instituto Nacional de Neurología y Neurocirugía Manuel Velasco Suárez (INNNMVS)' stroke clinic in Mexico City, in which consecutive patients with any type of stroke assessed in the emergency department (ED) from January to June 2019 were enrolled. Our institution is a referral public hospital that provides medical care to adults with neurological diseases from Mexico City and surrounding rural areas.

The population consisted of patients who arrived at the ED in the first 6 months of 2019 and were diagnosed with any type of acute stroke within the study protocol. Stroke subtypes included were IS, transient ischemic attack (TIA), intracerebral hemorrhage (ICH) and cerebral venous thrombosis (CVT). We excluded subarachnoid hemorrhage because of logistical reasons since these strokes were treated by neurosurgery in our hospital. Trauma was also excluded.

We collected demographic data (including age, gender, rural or urban living conditions, level of education), prehospital notification, the onset-to-door (OTD) time of any type of stroke (defined as the time elapsed from the first neurological symptoms detected until arrival at the ED), and baseline stroke severity measured on the National Institute of Health Stroke Severity Scale (NIHSS). Additionally, we included door-to-needle (DTN) time for IS patients. Information about hospital evolution (clinical, laboratory and imaging data) and functional outcomes (NIHSS at discharge and mRs at 3 months) was obtained. Additionally, a telephone interview was conducted with patients or a responsible family member to find out the reason they considered the most important for the delay in arrival at our center since the onset of stroke.

For statistical analysis, the SPSS 25.0 package (IBM SPSS Statistics for MacOs, Armonk, NY, USA: IBM Corp.) was used. We performed a descriptive analysis of the variables mentioned, which were expressed as medians, proportions and ranges. Anonymity was maintained and information on specific patients will not be disclosed, only as clustered variables. This study does not require informed consent due to its retrospective nature, the observational design using mainly patient file information, and because there were no additional tests or therapeutic interventions to those that the patient requires as part of their medical care (based on Regulation of the General Health Law on Health Research: Second Title, Chapter I, Article 17, Section I, research without risk, does not require informed consent, observational study).

## Results

We analyzed 116 patients with acute stroke who were evaluated in the ED January–June 2019. The mean age of the population was 65 ± 15 years, 67 (57.8%) were men and 57 (49.1%) had ≤ 6 years of education (Table 1).

**Table 1:** Demographic data and prehospital characteristics of patients arriving at the ED with any type of stroke (*n* = 116).

	***n* (%)**
Age	65 ± 15 (range 24–97)
**Gender**	
Men	67 (57.8%)
Women	49 (42.2%)
**Education**	
Illiterate	15 (12.9%)
Elementary school	42 (36.2%)
Middle school	26 (22.4%)
High school	11 (9.5%)
Technical	5 (4.3%)
University degree	17 (14.7%)
**Residence**	
Urban	107 (92.2%)
Rural	9 (7.8%)
**No. of consultations before arrival**	
0	57 (49.1%)
1	27 (23.3%)
2	20 (17.2%)
3	9 (7.8%)
4	3 (2.6%)
**Patient transportation**	
Ambulance	19 (16.4%)
Other	97 (83.6%)
**Prehospital notification system**	
Yes	4 (3.4%)
No	112 (96.6%)

*ED, emergency department*.

Most of the patients came from urban housing (*n* = 107, 92.2%). Fifty-nine (50.9%) patients went to other hospitals prior to arriving at INNNMVS, of which 27 (23.3%) went to one hospital, 20 (17.2%) went to two hospitals, 9 (7.8%) went to three hospitals and 3 (2.6%) to four hospitals. Additionally, 83 (71.6%) patients arrived at our hospital in a private car, 19 (16.4%) in an ambulance, 12 (10.3%) in a taxi and 2 (1.7%) in public transport. Regarding prehospital stroke care, we received prenotification in only 4 (3.4%) cases; 3 of these patients were transferred *via* ambulance, and another came by private car (Table 1).

**Figure 1:**
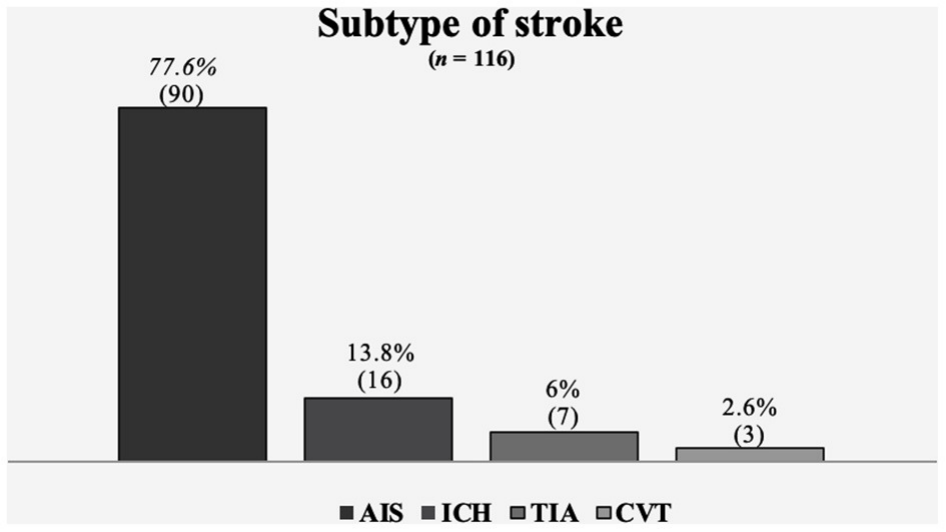
Subtype of stroke. AIS, acute ischemic stroke; ICH, intracerebral hemorrhage; TIA, transient ischemic attack; CVT, cerebral venous thrombosis.

Regarding subtype of stroke, 90 (77.6%) patients were diagnosed with ischemic stroke, 7 (6%) transient ischemic attack, 16 (13.8%) intracerebral hemorrhage and 3 (2.6%) with cerebral venous thrombosis (Figure 1). In the IS, as classified by TOAST, 38 (42.2%) patients experienced a stroke of undetermined etiology, 13 (14.4%) cardioembolic stroke, and 11 (12.2%) large artery atherosclerosis. In patients with hemorrhagic stroke, 11 (68.8%%) experienced a hypertensive stroke, 4 (25%) undetermined type and 1 (6.2%%) had a structural cause. The mean OTD time for all patients was 17 h (range 45 min−10 days), and the time between arrival to the ED and brain imaging for initial diagnosis was 28 ± 12 min. Overall, 113 (97.4%) patients received a simple or vascular cranial tomography (CT) scan as the initial brain imaging study, and in 49 (42.2%) patients, magnetic resonance imaging (MRI) was performed as the first or subsequent diagnostic study. The baseline NIHSS score was 9 ± 6 in all types of stroke and 5 ± 5 at discharge. Eight (6.9%) patients were hospitalized in the intensive care unit. There were 3 deaths (2 IS and 1 ICH), in whom the initial NIHSS was between 19 and 25 points.

In acute ischemic stroke, only 32 (35.5%) arrived at the hospital within a 4.5 h therapeutic window, and 16 (17.8%) received intravenous thrombolysis, with a mean DTN time of 37 ± 10 min (Table 2).

**Table 2:** Patient characteristics at admission and outcome for any type of stroke and for patients with AIS subtype.

**Any type of stroke**	
Baseline NIHSS	9 ± 6
Mean OTD time	17 h (45 min−10 d)
Time to imaging	28 ± 12 min
mRs at 90 d	2 ± 1.5
Physical therapy program at 90 d	41 (35.3%)
Current neurology follow-up	49 (42.2%)
AIS	
OTD time < 4.5 h	35.5% (32/90)
IVT	17.8% (16/90)
DTN time	37 ± 10 min

*AIS, acute ischemic stroke; NIHSS, National Institute of Health Stroke Scale; OTD, onset-to-door; mRs, modified Rankin scale; IVT, intravenous thrombolysis; DTN, door-to-needle*.

Telephone interviews were conducted with 61 patients or a responsible family member. In 55 (47.4%) cases, the primary caregiver could not be contacted to obtain this information. When asked about the main reason for arrival delay to the ED at INNNMVS, 26 (42.6%) patients mentioned going to a different health facility first (either private medical office or a hospital), 21 (34.4%) patients/primary caregivers said they did not recognize stroke symptoms and did not know about treatment urgency, 6 (9.8%) were far from INNNMVS when symptom onset began, 4 (6.6%) patients did not have access to a fast means of transport, and 4 (6.6%) reported a long wait time after calling for an ambulance (Table 3).

**Table 3:** Perception of patient/primary caregiver about the reasons for the delay in arriving to the ED at the INNNMVS after the onset of symptoms (*n* = 61).

Evaluation in other facilities prior to arrival at our center	26 (42.6%)
Ignorance of stroke symptoms or treatment urgency	21 (34.4%)
Far from INNNMVS when symptom onset began	6 (9.8%)
Not access to a fast means of transport	4 (6.6%)
Long wait time after calling for an ambulance	4 (6.6%)

*ED, emergency department; INNNMVS, Instituto Nacional de Neurología y Neurocirugía Manuel Velasco Suárez*.

In terms of outcomes and post-stroke care, the mRs at 3 months was 2 ± 1.5 for all patients studied; only 41 (35.3%) patients received physical rehabilitation at discharge, and less than half of patients (*n* = 49, 42.2%) were followed up by a neurologist.

## Discussion

This study describes barriers to optimal acute management of stroke. Multiple medical evaluations prior to arrival at a tertiary hospital were the main prehospital barrier to optimal acute management of stroke in a developing country, who mostly arrived in a private car and without prenotification. Prior multiple medical evaluations and the lack of knowledge about stroke symptoms or the urgency of treatment are the barriers most frequently cited by patients and their family members. In half of the cases evaluated, the education level was ≤ 6 years, which may have influenced the decision to seek prompt medical care, as reported in Brainin's review, where only 27% of patients who arrived at a stroke service in a tertiary hospital were aware that they had experienced a stroke (7). Previous studies have shown that recognition of stroke symptoms by educational efforts reduces OTD time in IS (9, 10). In LMICs such as Mexico, it is necessary to improve education on the recognition of stroke symptoms and the importance of seeking immediate medical evaluation.

Although public education about stroke plays a fundamental role, we observed that only 16% of patients arrived at the ED by ambulance, the majority came by private car, and our hospital was pre-notified in only 4 out of 116 cases. These results are similar to those reported in a systematic review about stroke care in LMICs, where the authors concluded that ambulance services are underutilized for stroke patients and that training paramedics to recognize stroke, pre-notify hospitals and take patients to a center with brain imaging availability can reduce time lost in transit and investigations in the ED (11). Moreover, if the patient is taken to a hospital with basic imaging equipment but no stroke team, linking the hospital to a tertiary center via telestroke will enable better diagnosis and prompt treatment (12).

It is important to note that 50% of patients went to other health facilities (range 1–4) prior to their arrival at the INNNMVS; although we did not study the reasons for these multiple evaluations, it is possible that patients/primary caregivers and first medical contacts did not know to refer or transport the patient to a facility equipped to handle a patient with an acute stroke. This finding may have a negative impact on patients' functional outcomes, since it has been observed that rapid and precise diagnosis are effective components in improving overall outcomes in stroke patients (7).

These missteps could have had an impact on the mean OTD time, which was 17 h (45 min-10 days) in the total sample. As a consequence, only 35% of all acute ischemic strokes arrived within the 4.5 h time window for thrombolysis, leaving more than half of the patients with acute IS out of reperfusion treatment. Nevertheless, it was possible to thrombolize 17.8% of the patients with a mean DTN time of 37 ± 10 min, a percentage higher than that previously reported in the Mexican health system (7.6%) and with shorter thrombolysis times (previous mean DTN time 81 ± 51 min) (5); these findings may be secondary to greater experience in our stroke center, which may not reflect reality in the rest of the country. In previous studies, the main barrier to optimal stroke treatment, according to the health professionals, was health staff capacity (especially lack of a stroke team) (6), a factor that we do not consider to have a great influence in our tertiary hospital but, as previously mentioned, may not represent the situation throughout Mexico. In our study, in-hospital barriers were not evaluated, but we are sure that there are a lot of that also requires attention to optimal stroke treatment.

Although all stroke patients in our hospital were sent to a rehabilitation clinic at discharge, when interviewed, only 35% had received physical rehabilitation after stroke, and <50% had a follow-up visit with a neurologist. Since it was not the purpose of the study, we did not fully explore the reasons for these findings. However, we recommend that patients receive timely care both at baseline and at follow-up, which will have an impact on long-term functional outcomes.

Improving stroke care networks is a difficult job in LMICs, but not impossible; the best example is the *Brazilian Stroke Project*, which resulted in an increase from 35 stroke centers in Brazil in 2008 to 149 in 2017 and a drop in mortality from 17.9% in 2010 to 12.8% in 2014 (13).

The main limitation of our study is that although the patients were prospectively included in the database, the analysis was retrospective, but our data was obtained by a stroke neurologist. Phone interviews with the patient or primary caregiver were performed 1 year after the stroke, which may skew some of the information obtained, however, although this result is not the main conclusion of our study, it provides an idea of the family perception of a patient with acute stroke. We also recognize that our hospital is a tertiary neurological center in one of the main cities in the country, and barriers to care probably are lower than those found in the rest of the country (mainly in rural areas) or in other regions of Latin America. Thus, future studies should explore barriers to acute stroke care in other regions with greater public health problems, with the support of a standard stroke literacy questionnaire.

This study provides important data about the prehospital barriers to timely medical management in patients with acute stroke in a developing country. These data show the improvements in public health that could be made in Mexico and in other countries with similar socioeconomic conditions, promoting progress in stroke care.

In conclusion, in this small, retrospective, single center study, the main prehospital barrier to optimal acute management of stroke in a developing country is multiple medical evaluations prior to the patient's transport to a specialized stroke hospital, who mostly arrived in a private car and without prenotification. Patient's family considers that prior multiple medical evaluations and the lack of knowledge of stroke symptoms and treatment urgency were the main reasons for the delay in arrival at the INNN, but this result must be corroborated in prospective studies with larger samples. These barriers can be overcome by strengthening public education and improving patient transfer networks and telemedicine.

## Data Availability Statement

The raw data supporting the conclusions of this article will be made available by the authors, without undue reservation.

## Ethics Statement

Ethical review and approval was not required for the study on human participants in accordance with the local legislation and institutional requirements. Written informed consent for participation was not required for this study in accordance with the national legislation and the institutional requirements.

## Author Contributions

All authors listed have made a substantial, direct, and intellectual contribution to the work and approved it for publication.

## Conflict of Interest

The authors declare that the research was conducted in the absence of any commercial or financial relationships that could be construed as a potential conflict of interest.

## Publisher's Note

All claims expressed in this article are solely those of the authors and do not necessarily represent those of their affiliated organizations, or those of the publisher, the editors and the reviewers. Any product that may be evaluated in this article, or claim that may be made by its manufacturer, is not guaranteed or endorsed by the publisher.
